# In Vitro and In Vivo Studies of Biodegradability and Biocompatibility of Poly(εCL)-*b*-Poly(EtOEP)-Based Films

**DOI:** 10.3390/polym12123039

**Published:** 2020-12-18

**Authors:** Ilya Nifant’ev, Andrey Shlyakhtin, Pavel Komarov, Alexander Tavtorkin, Evgeniya Kananykhina, Andrey Elchaninov, Polina Vishnyakova, Timur Fatkhudinov, Pavel Ivchenko

**Affiliations:** 1 Chemistry Department, M.V. Lomonosov Moscow State University, 1-3 Leninskie Gory, 119991 Moscow, Russia; shlyahtinav@mail.ru (A.S.); phpasha1@yandex.ru (P.I.); 2 A.V. Topchiev Institute of Petrochemical Synthesis RAS, 29 Leninsky Pr., 119991 Moscow, Russia; komarrikov@yandex.ru (P.K.); tavtorkin@yandex.ru (A.T.); 3 Faculty of Chemistry, National Research University Higher School of Economics, 20 Miasnitskaya Str., 101000 Moscow, Russia; 4 Research Institute of Human Morphology, 3 Tsyurupy St., 117418 Moscow, Russia; e.kananykhina@gmail.com (E.K.); tfat@yandex.ru (T.F.); 5 National Medical Research Center for Obstetrics Gynecology and Perinatology Named after Academician V.I. Kulakov of Ministry of Healthcare of Russian Federation, 4 Oparina Str., 117997 Moscow, Russia; elchandrey@yandex.ru (A.E.); vpa2002@mail.ru (P.V.); 6 Department of Histology, Cytology and Embryology, Peoples’ Friendship University of Russia, Miklukho‐Maklaya 6 Str., 117198 Moscow, Russia

**Keywords:** polyesters, polyphosphoesters, polycaprolactone, protein adsorption, ring-opening polymerization, cytotoxicity, immunohistochemical test

## Abstract

The control of surface bioadhesive properties of the subcutaneous implants is essential for the development of biosensors and controlled drug release devices. Poly(alkyl ethylene phosphate)-based (co)polymers are structurally versatile, biocompatible and biodegradable, and may be regarded as an alternative to poly(ethylene glycol) (PEG) copolymers in the creation of antiadhesive materials. The present work reports the synthesis of block copolymers of ε-caprolactone (εCL) and 2-ethoxy-1,3,2-dioxaphospholane-2-oxide (ethyl ethylene phosphate, EtOEP) with different content of EtOEP fragments, preparation of polymer films, and the results of the study of the impact of EtOEP/εCL ratio on the hydrophilicity (contact angle of wetting), hydrolytic stability, cytotoxicity, protein and cell adhesion, and cell proliferation using umbilical cord multipotent stem cells. It was found that the increase of EtOEP/εCL ratio results in increase of hydrophilicity of the polymer films with lowering of the protein and cell adhesion. MTT cytotoxicity test showed no significant deviations in toxicity of poly(εCL) and poly(εCL)-*b*-poly(EtOEP)-based films. The influence of the length of poly(EtOEP)chain in block-copolymers on fibrotic reactions was analyzed using subcutaneous implantation experiments (Wistar line rats), the increase of the width of the fibrous capsule correlated with higher EtOEP/εCL ratio. However, the copolymer-based film with highest content of polyphosphate had been subjected to faster degradation with a formation of developed contact surface of poly(εCL). The rate of the degradation of polyphosphate in vivo was significantly higher than the rate of the degradation of polyphosphate in vitro, which only confirms an objective value of in vivo experiments in the development of polymer materials for biomedical applications.

## 1. Introduction

The prevention of unspecific protein adsorption and cell interactions, which can lead in vivo to a foreign body reaction, is important in developing new biomaterials which are in direct contact with body tissues, such as implants, prosthetics, biosensors, controlled drug release devices [1]. Initial nonspecific protein adsorption on the surface of the implant supports the formation of fibrous capsule, a diffuse barrier for drug release and blood penetration to biosensors [2]. The development of the materials with anti-adhesive properties is of great relevance. Since poor protein resistance is often associated with surface hydrophobicity, efficient way to such materials is a modification of the surface by poly(ethylene glycol) (PEG) [1,3,4]. However, low biodegradability of PEG causes formation of PEG antibodies, emergence of hypersensitivity, etc. [5]. In some experiments with subcutaneous injection of PEGylated implants, the formation of fibrous capsule was detected [6]. Consequently, the search of PEG alternative for ‘hydrophilization’ of the surface of the implants is still relevant for the chemistry of biodegradable polymers.

**Scheme 1:**
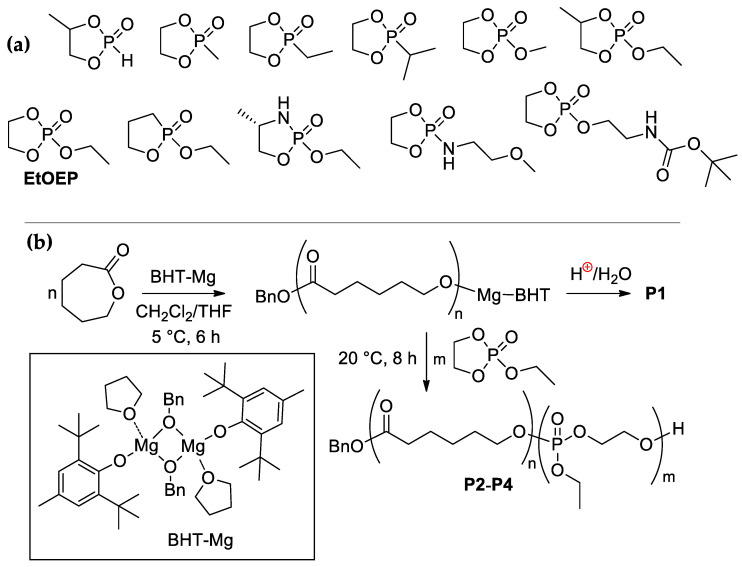
(**a**) Cyclic phosphorus-containing monomers suitable for the synthesis of water-soluble biodegradable polymers [5]; (**b**) Synthesis of poly(εCL) and poly(εCL)-*b*-poly(EtOEP).

Ethylene phosphates, phosphonates and phosphoramidates with short alkyl substituents (Scheme 1a) represent promising cyclic substrates for the synthesis of copolymers with hydrophilic biodegradable blocks using catalytic ring-opening polymerization (ROP, Scheme 1b) [5,7,8,9,10,11,12,13,14]. 2-Ethoxy-1,3,2-dioxaphospholane 2-oxide (ethyl ethylene phosphate, EtOEP) is one of the most reliable monomers for the preparation of hydrophilic polyphosphoesters (PPEs) due to high reactivity and low tendency to form branched polymers [15,16,17]. Block copolymers poly(εCL)-*b*-poly(EtOEP) were synthesized previously using tin (II) octanoate as a catalyst [15].

The main part of the works on biomedical application of PPEs and related polymers is related to drug and gene delivery [7,9,10,12,18,19,20,21,22], there were only few studies dealing with the use of PPEs for the surface modification [5,23,24]. As far as we know, the influence of the surface modification by PPEs on immune response have not been studied to date.

Taking into account possible toxicity of tin (II) derivatives [25], it seems preferable to use coordination catalysts, based on ‘biometals’ (Na, Mg, Ca, Al, Zn), in the preparation of polymers for biomedical applications. In the present paper, the synthesis of εCL homopolymer **P1** and block-copolymers poly(εCL)-*b*-poly(EtOEP) **P2**–**P4** with *DP*_n_(εCL) ~200 and different εCL/EtOEP ratios (~32, ~8 and ~3, respectively) by living ROP of εCL and EtOEP, initiated by non-toxic complex [(BHT)Mg(μ-OBn)(THF)]_2_ (BHT-Mg) [26] (Scheme 1b) followed by the preparation of polymer films, is reported. These films were studied in vitro with a view to evaluating the impact of EtOEP/εCL ratio on the hydrophilicity, hydrolytic stability, cytotoxicity, protein and cell adhesion, and cell proliferation using umbilical cord multicomponent stem cells (UC MSCs). For the first time for PPE-containing materials, in vivo subcutaneous implantation experiments using Wistar rats were performed.

## 2. Materials and Methods

### 2.1. Synthesis of (co)Polymers

#### 2.1.1. General Experimental Remarks

All of the synthetic and polymerization experiments were performed under a purified argon atmosphere. CH_2_Cl_2_ was washed with aqueous Na_2_CO_3_, stirred with CaCl_2_ powder, refluxed over CaH_2_ for 8 h and distilled. Tetrahydrofuran (THF) and diethyl ether (Et_2_O) (Merck, Darmstadt, Germany) were refluxed with Na/benzophenone and distilled prior to use. εCL (Merck, Darmstadt, Germany) was distilled prior to use under argon over CaH_2_. Ethyl ethylene phosphate (EtOEP) [27] and BHT-Mg [26] were synthesized according to the literature procedures.

CDCl_3_ (D 99.8%, Cambridge Isotope Laboratories, Inc., Tewksbury, MS, USA) was distilled over P_2_O_5_ and stored over 4 Å molecular sieves. The ^1^H (400 MHz) and ^31^P (162 MHz) NMR spectra were recorded on a Bruker AVANCE 400 spectrometer (Bruker, Billerica, MS, USA) at 20 °C. The chemical shifts were reported in ppm relative to the solvent residual peak (*δ* = 7.26 ppm).

Size exclusion chromatography (SEC) was performed on an Agilent PL-GPC 220 chromatograph (Agilent Technologies, Santa Clara, CA, USA) equipped with a PLgel column, using THF as an eluent (1 mL/min). The measurements were recorded with universal calibration according to polystyrene standards at 40 °C.

#### 2.1.2. Synthesis of Poly(εCL) **P1**

εCL (3.82 mL, 34.5 mmol) was placed into flame-dried vial, equipped with magnetic stirrer bar and septum. CH_2_Cl_2_ (11.7 mL) was added, the solution was cooled to 5 °C, the solution of BHT-Mg (73 mg, 0.17 mmol) in THF (2.0 mL) was added. After 6 h of stirring at 5 °C, AcOH (52 µL) was added. The solvents were removed under reduced pressure, the residue was dried in vacuo, dissolved in CH_2_Cl_2_ (60 mL). The solution of poly(εCL) was washed by 1M HCl (30 mL), distilled water (2 × 30 mL), dried over MgSO_4_, and filtered. The solution was evaporated and poured into Et_2_O. The yield was 2.45 g (62%).

End-group analysis of ^1^H NMR spectra of **P1** (see Figure S1 in the Supplementary Materials) was used for the determination of *M*_n_^NMR^ by comparative integration of the signals of poly(εCL) protons and aromatic protons of benzyl group at 7.3–7.4 ppm.

#### 2.1.3. Synthesis of Poly(εCL)-*b*-Poly(EtOEP) **P2**–**P4**

εCL (3.82 mL, 34.5 mmol) was placed into flame-dried vial, equipped with magnetic stirrer bar and septum. CH_2_Cl_2_ (11.7 mL) was added, the solution was cooled to 5 °C, the solution of BHT-Mg (73 mg, 0.17 mmol) in THF (2.0 mL) was added. After 6 h of stirring at 5 °C, calculated qualities of EtOEP (20, 40 and 60 equivalents relatively to BHT-Mg, 410, 820, and 1230 µL for the synthesis of **P2**, **P3** and **P4**, respectively) were added, the reaction mixtures were stirred for 10 min at 5 °C. Then, AcOH (52 µL) was added. The solvents were removed under reduced pressure, the residue was dried in vacuo, dissolved in CH_2_Cl_2_ (80 mL). The solutions were shaken with 1M HCl, the emulsions were evaporated under reduced pressure, polymer residues were separated by filtration, and dissolved in CH_2_Cl_2_. This procedure was repeated with H_2_O. The polymers obtained were dried at 0.02 Torr to constant weight. The residues were dissolved in dimethoxymethane (minimal amount) and poured into Et_2_O with a separation of the polymer precipitates. This procedure was repeated once. The products were dried in vacuo and analyzed. The yields were 2.94 g (66%) for **P2**, 2.89 g (58%) for **P3**, 2.86 g (52%) for **P4**.

*M*_n_^NMR^ was determined by comparative integration of the signals of poly(εCL) protons, poly(EtOEP) protons and aromatic protons of benzyl group in ^1^H NMR spectra of the copolymers (see Figures S2–S4 in the Supplementary Materials).

### 2.2. Preparation and Mechanical Testing of Polymer Films

Polymer films for mechanical testing **FM1**–**FM4** (film thickness 0.16–0.17 mm) were prepared using HLCL-1000 hot melt coater/laminator (ChemInstruments, Fairfield, OH, USA). Dog-bone tensile specimens (ASTM standard D1708-96, 22 × 5 mm) were prepared by punching the films from a stainless steel die. A I1140M-5-01-1 universal tensile testing machine (Tochpribor-KB, Ivanovo, Russia) and ASTM D638 method were used for film mechanical testing.

### 2.3. Preparation of Polymer Films for Hydrolytic and Biomedical Testing

#### 2.3.1. Preparation of Polymer Films

Polymer films for biomedical testing **FB1**–**FB4** were prepared by dissolution of 300 mg of polymers **P1**–**P4** in CH_2_Cl_2_ (4 mL), followed by slow evaporation of the solution in Petri dishes (6 cm diameter). Copolymer **P4** was also used in preparation of the sample **FB4′** with higher film thickness by dissolution of 1.00 g of **P4** in CH_2_Cl_2_ (6 mL) followed by slow evaporation in Petri dish (6 cm diameter).

#### 2.3.2. Contact Angle Measurements

For the determination of hydrophilicity of the films, the static contact angle of distilled water on the surface of the films **FB1**–**FB4** was measured using a LK-1 goniometer equipped with a CCD camera (RPC OpenScience Ltd., Krasnogorsk, Russia) for both surfaces, smooth (film side that was in contact with glass) and rough (film side that was in contact with air during evaporation of the polymer solution). The images of water drops on the sample surface were analyzed with software supplied by the manufacturer. Ten samples were measured in each type of the films. Initially, distilled water (5 mL) was used in each measurement after exposure for 3 s at ambient temperature and 70% relative humidity.

#### 2.3.3. Hydrolytic Degradation in Vitro

Huber MPC-E immersion thermostat (Huber Kältemaschinenbau, Offenburg, Germany) was used in experiments on hydrolytic polymer degradation in PBS that were performed by the common method [28,29]. The temperature of hydrolysis was 39 °C (normal temperature of the rat body, to compare with the results of the experiments in vivo). Given the stability of poly(εCL) blocks against degradation under mild conditions, the residual content of poly(EtOEP) fragments was analyzed using ^1^H NMR spectroscopy.

Scanning electron microscope (SEM) images of the polymer surfaces were obtained using a JEOL JSM-6000PLUS Neoscope II (Jeol Ltd., Tokyo, Japan) at the accelerating voltage of 15.0 kV.

#### 2.3.4. Preparation of the Samples for Biomedical Testing

Phosphate-buffered saline (PBS) containing 0.131 mol/L NaCl and 0.0027 mol/L KCl (Merck, Darmstadt, Germany) was used as purchased. For biomedical studies, the samples of **FB1**–**FB4** and **FB4′** were placed to 96% and 70% ethanol (20 min exposition in each solution) for wetting and sterilization, ethanol was removed by exposition in PBS (3 × 5 min). Finally, the film samples were exposed for 1 h in culture medium.

### 2.4. Protein Adhesion

The samples of **FB1**–**FB4** were incubated for 18 h at 4 °C in PBS containing green fluorescent protein (GFP; Evrogen, Moscow, Russia) with a concentration of 0.2 mg/mL, washed three times by PBS and studied using a Leica DM 4000 fluorescent microscope (Leica Microsystems GmbH, Wetzlar, Germany).

### 2.5. Cultivation of UC MSCs

Based on the data compiled and reported earlier [30,31], UC MSCs were isolated using fermentation from Wharton’s jelly of umbilical cord. Collecting of umbilical cord was approved by the Commission of Biomedical Ethics at National Medical Research Center for Obstetrics, Gynecology and Perinatology of Ministry of Healthcare of Russian Federation, Moscow (ethics committee approval protocol no. 12, 17 November 2016). Written informed consent was obtained from all participants prior to the study. UC MSCs were cultured in Dulbecco’s Modified Eagle Medium F-12 (DMEM-F12) (PanEco, Moscow, Russian Federation) containing 10% fetal bovine serum (FBS; PAA Laboratories, Linz, Austria) at 37 °C under 5% CO_2_ humidified atmosphere. Belonging of the cell culture to MSCs was confirmed by the estimation of the expression of positive (CD90, CD105) and negative (CD34, CD45) markers as well as possibility of induced cell differentiation in osteogenic, chondrogenic and adipogenic directions in vitro.

UC MSCs were seeded to the surface of film samples and cultured at 37 °C under 5% CO_2_ humidified atmosphere. The samples (three for each type of polymer **FB1**–**FB4**) were transferred to bioreactor vials (SPL Life Sciences, Pocheon, Korea) containing 5 mL of cell suspension (8 × 10^5^ cells total). The vials were placed into orbital shaker (BioSan, Riga, Latvia), placed to CO_2_ incubator. Cell cultivation was carried out during 24 h at 75 rpm and then during 48 h at 50 rpm.

### 2.6. Cell Visualization and Count

For cell visualization and cell count on the surface of polymer films, UC MSCs seeded samples were fixed in 4% paraformaldehyde at 0 °C, then the cell nuclei were stained with DAPI (Merck, Darmstadt, Germany) following the manufacturer’s protocol.

Additionally, to observe the cell morphology on the surface of the films, the cells were labeled with a fluorescent red-orange vital dye PKH26 (Merck, Darmstadt, Germany) before settling the samples, according to manufacturer’s recommendations.

### 2.7. Cytotoxicity Studies

Quantitative assessment of the cytotoxic properties of the films was obtained using a standard MTT test with an incubation period of one, two, and four days. UC MSCs were seeded to the surface 96-well culture plate at a density of 7.0 × 10^3^ cells/well (70% confluence) and cultured in growth medium at 37 °C under 5% CO_2_ humidified atmosphere. Then 20 µl MTT (Merck, Darmstadt, Germany; 5 mg/mL) was added to each well of the plate and the plates were incubated at 37 °C for additional 2 h. The supernatants were removed, and 50 µL DMSO was added to each well. Absorbance was measured at λ = 570 nm on a Multiskan GO Spectrophotometer (Thermo Fisher Scientific, Waltham, MS, USA).

### 2.8. Immunocytochemical Studies

UC MSCs seeded samples were treated with Hanks’ Balanced Salt solution (Merck, Darmstadt, Germany), fixed by 4% paraformaldehyde (5 min) and by cold methanol (1 min), and stained with antibodies ab15580 (Abcam, Cambridge, UK) against proliferation marker Ki-67 according to manufacturer’s recommendations. The second type of antibodies was PE-conjugated sc3739 (Santa Cruz Biotechnology, Dallas, TX, USA), cell nuclei were stained with DAPI (Merck, Darmstadt, Germany). The observations were carried out with the use of a Leica DM 4000 B fluorescent microscope and LAS AF v.3.1.0 build 8587 software (Leica Microsystems GmbH, Wetzlar, Germany).

### 2.9. Subcutaneous Implantation Experiments

#### 2.9.1. Animals

Outbred, eight-week-old male Wistar rats (250–300 g) were obtained from the Institute for Bioorganic Chemistry branch animal facilities (Pushchino, Moscow, Russia). All experimental work involving animals was carried out according to the Standards of Laboratory Practice (National Guidelines No. 267 by Ministry of Healthcare of the Russian Federation, 1 June 2003), and all efforts were made to minimize suffering. The animals were adapted to laboratory conditions (23 °C, 12 h/12 h light/dark, 50% humidity, ad libitum access to food and water) for two weeks prior to manipulation.

#### 2.9.2. Subcutaneous Administration of the Polymer Films

**Figure 1:**
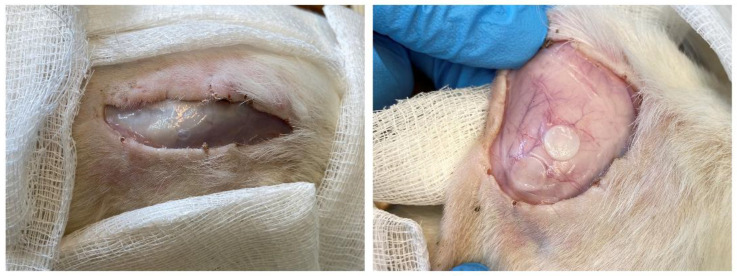
Subcutaneous administration of the polymer films.

All manipulations with animals were carried out in accordance with ‘Rules for carrying out work using experimental animals’ (order of the USSR Ministry of Health No. 755 dated 12.08.1977) after approval by Ethical Review Board at the Scientific Research Institute of Human Morphology (Protocol no. 10, 4 October 2019). The experiment was performed using 28 male rats of the Wistar line (mass of body 200–250 g). The animals were anesthetized using an intramuscular injection of zoletil (at the rate of 10 mg/kg) and meditin (at the rate of 0.12 mg/kg). In rats, hair was shaved off in the interscapular area, fixed on the operating table, and the operating field was treated with 70% alcohol. With scissors, a skin incision was made in the interscapular region, pockets were made bluntly on each side of the incision under the skin, where the sample of the polymer film (0.7 cm diameter) was inserted, the skin was sutured with interrupted sutures (Figure 1), and the wound was treated with 70% alcohol. By the number of the films under study (**FB1**–**FB4** and **FB4′**), the rats were divided into five groups, containing four rats for each **FB1**–**FB4** and **FB4′** implants.

#### 2.9.3. Morphometric Studies

After 14 days (**FB4** and **FB4′** implants) and 28 days (all implants) the rats were taken out from the experiment by euthanasia in a CO_2_-chamber. The capsule with the implant was excised, and fixed in 10% formaldehyde solution within 72 h. The tissue samples were encased in paraffin. To study morphological changes, cross sections of 7 μm thick were made using rotary microtome Accu-Cut SRM (Sakura Finetek, Tokyo, Japan). The sections were stained by hematoxylin and eosin, dehydrated and enclosed in a synthetic mounting medium (BioVitrum, Saint-Petersburg, Russia).

Morphometric studies were carried out on the micrographs of the samples, stained by hematoxylin and eosin, using a Leica DM 2500 microscope and ImageScope M software (Leica Microsystems GmbH, Wetzlar, Germany) (Figure S5 in the Supplementary Materials). For each experiment, six sections were randomly selected and micrographs were made for 10 fields of view on each histological section.

#### 2.9.4. Immunohistochemical Studies

A part of the samples was fixed using liquid nitrogen. Cryosections of 5–7 μm thickness were made using cryotom Leica CM1900 (Leica Microsystems GmbH, Wetzlar, Germany), and SuperFrost glass slides (Menzel, Germany). The sections were stained with ab125212 antibodies (Abcam, Cambridge, UK) against the macrophage marker CD68; cell nuclei were stained by DAPI. To estimate the quantity of CD68+ cells, micrographs at 400× magnification (Leica DM 4000 B microscope) were analyzed.

### 2.10. Statistical Analysis

The data is presented as the mean  ±  standard deviation (SD) and median with interquartile range. One-way ANOVA followed by Dunnett’s multiple comparisons test was performed using Sigma Stat 3.5 (Systat Software, San Jose, CA, USA). Values of *p* < 0.05 were considered statistically significant.

## 3. Results and Discussion

### 3.1. Polymerization and (co)Polymer Characteristics

As was demonstrated previously, BHT-Mg catalyst is highly efficient in living ROP of cyclic substrates such as cyclic esters [26,32] and CEPMs [11,33,34,35,36,37]. In our experiments (Table 1), εCL homopolymer **P1** and εCL/EtOEP block copolymers **P2**–**P4** were obtained using consecutive low-temperature polymerization of εCL and EtOEP. The living character of BHT-Mg initiated polymerization of εCL and EtOEP was proved by the consistency of εCL/EtOEP ratios in copolymers formed and in copolymers separated after a series of successive re-precipitations. These data were obtained by the analysis of NMR spectra of the reaction mixtures and copolymers after separation (see Section S1 in the Supplementary Materials).

### 3.2. Mechanical Properties of Polymers

The tensile strength properties of the films **FM1**–**FM4** were examined at 25 °C using standard method (see Section 2.2), and the results are illustrated in Table 2. Poly(εCL) film **FM1** demonstrated highest tensile strength and elongation, these parameters showed a downward trend with an increasing of EtOEP content in block copolymers, in the transition from **FM2** to **FM4**. At the same time, **FM2** had the highest Young’s modulus among polymers studied. Based on mechanical test, copolymer film **FM4** with the highest EtOEP content must be recognized as not usable for the producing of biopolymer scaffolds.

### 3.3. Hyrdophilicity and Hydrolysis In Vitro

#### 3.3.1. The Results of the Contact Angle Measurements

**Figure 2:**
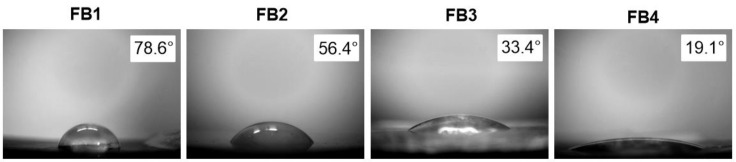
Contact angles of wetting for **FB1**–**FB4**.

As was to be expected, the εCL/EtOEP ratio has a direct impact on the contact angle values (Figure 2). Increasing of EtOEP content resulted in lowering of the contact angle; the films **FB3** and **FB4** can be considered as highly hydrophilic.

#### 3.3.2. Hydrolytic Degradation in Buffer Solution

**Figure 3:**
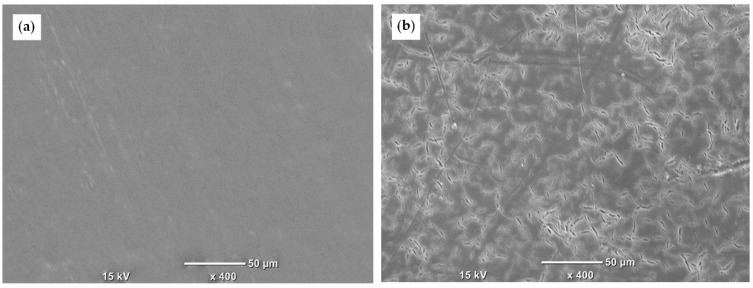
SEM images of the surfaces of polymer film **FB4** before (**a**) and after (**b**) 14 day hydrolysis in PBS.

To study in vitro hydrolytic degradation behavior we exposed 200 mg samples of **FB1**–**FB4** films with PBS at pH 7.4 and 37.0 ± 0.1 °C for 14 days. The morphologies of **FB4** films before and after hydrolysis were first characterized by SEM. Essential results were obtained for **FB4** film. The SEM results show that the topological structure of the **FB4** film before hydrolysis had no fibers and the surface was relatively smooth (Figure 3a). However, after 14 days the surface morphology has changed significantly with a formation of multiple caverns (Figure 3b).

**Figure 4:**
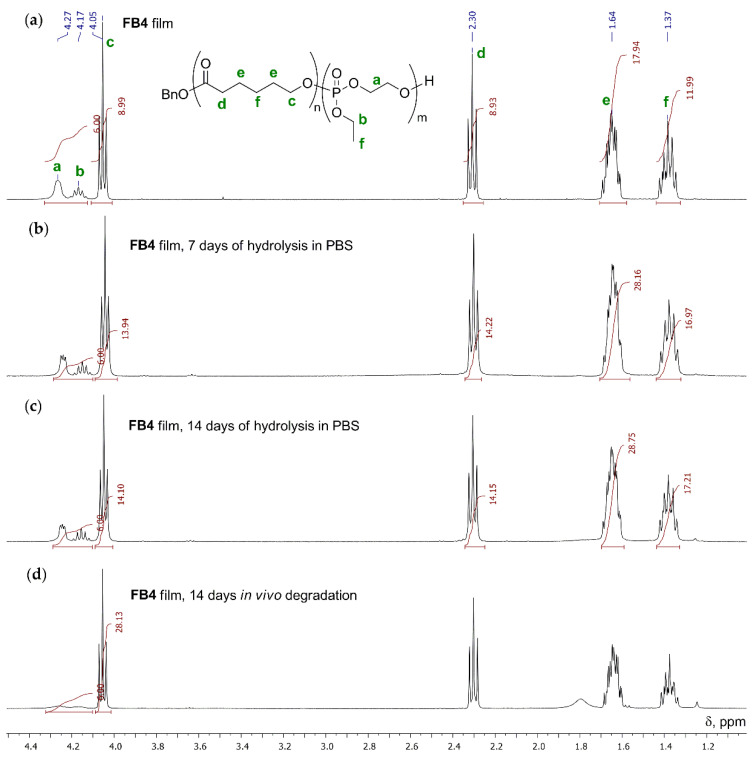
Hydrolytic degradation of **FB4** film (**a**) in vitro (**b**,**c**) and in vivo (**d**).

As was demonstrated recently by Wurm et al. [14], in aqueous solution poly(EtOEP) undergoes relatively slow hydrolysis, the major hydrolytic degradation pathway is backbiting with the participance of polyphosphate chain ends—P(O)(OEt)CH_2_CH_2_OH. For copolymers obtained, such a mechanism seems even more likely. In our experiments, within 7 days for **FB4** (Figure 4a) a marked decrease in EtOEP content was detected (Figure 4b), however, after additional seven days, the εCL/EtOEP ratio has changed little (Figure 4c). This can be attributed to superficial character of the hydrolysis affecting only poly(EtOEP) fragments, and lower hydrophilicity of the poly(εCL) surface formed.

### 3.4. Cytotoxicity

**Figure 5:**
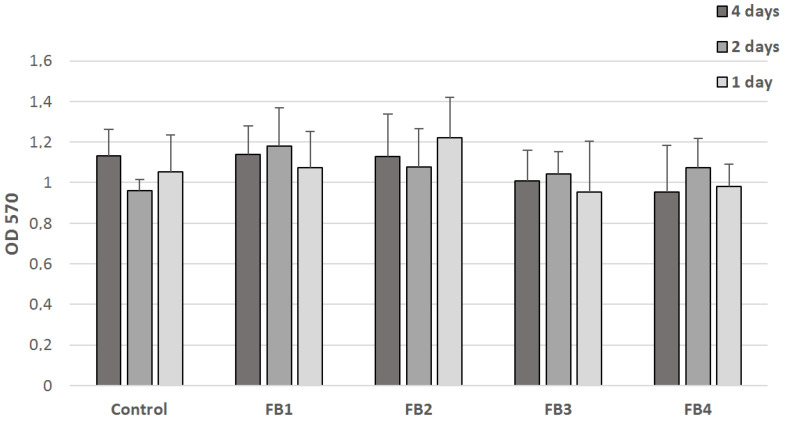
Evaluation of the cytotoxicity of polymer films **FB1**–**FB4** for UC MSCs: the results of MTT test after one, two, and four days of incubation.

Cell viability was assessed by the MTT assay based on the ability of live cells to convert the water-soluble yellow 3-(4,5-dimethylthiazol-2-yl)-2,5-diphenyltetrazolium bromide (MTT) into insoluble purple intracellular crystals of MTT-formazan. The conversion efficiency is indicative of the general level of dehydrogenase activity of the cells under study, which is to a certain extent directly proportional to the concentration of viable cells [38]. The cytotoxicity test was conducted on the UC MSCs using the complete media extracts of **FB1**–**FB4** films which were prepared by incubation of the film samples in the media containing 7.0 × 10^3^ cells/well, followed by extraction and registration of UV–VIS spectra. Figure 5 shows the absorption intensities at λ = 570 nm for all samples after one, two, and four days of incubation. No significant differences were found in the comparison of the absorption intensity values of the extracts from UC MSCs-loaded wells with polymer film samples and extract from UC MSCs-loaded well without an addition of polymers (blue dotted line). Comparison of cell viability within the same film sample at different time points revealed no significant difference between one, two, and four days. In this way, **FB1**–**FB4** demonstrated a complete absence of toxicity.

### 3.5. Protein Adhesion

**Figure 6:**
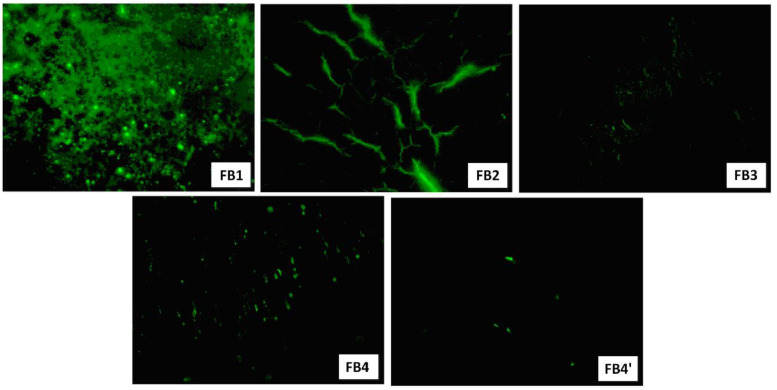
Adhesion of the GFP protein on the surface of the films **FB1**–**FB4** and **FB4′**. Fluorescent microscopy, magnification of 400×.

Protein adhesion is the process preceding cell adhesion. To compare the ability of the films **FB1**–**FB4** and **FB4′** to protein adhesion, the samples were incubated with GFP (see Section 2.4). Surface distribution of the adhered protein was analyzed using fluorescent microscopy (Figure 6). For poly(εCL) film **FB1** we observed uniform protein coverage. With the increasing of EtOEP content, protein adhesion decreased. It is noteworthy that for **FB2** with relatively high εCL/EtOEP GFP adhesion was uneven, exposing phosphate-free surface areas. For copolymer films with higher EtOEP content the cell adhesion was rare and spotty.

Therefore, even the minimal content of poly(ethylene phosphate) fragments in copolymer successfully prevent protein adhesion. For cell adhesion, we expected the same behavior.

### 3.6. Cell Adhesion and Cell Proliferation

**Figure 7:**
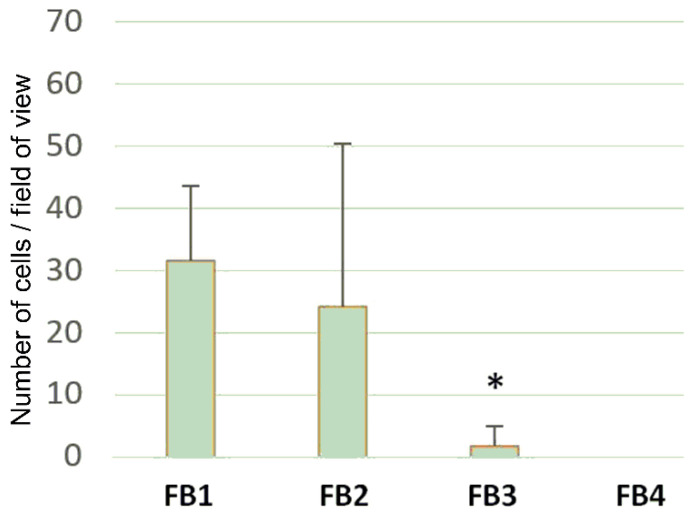
Evaluation of adhesive properties of polymer films **FB1**–**FB4** for UC MSCs (vertical scale—a number of cells in a field of view). * *p* < 0.05 in comparison with **FB-1**.

**Figure 8:**
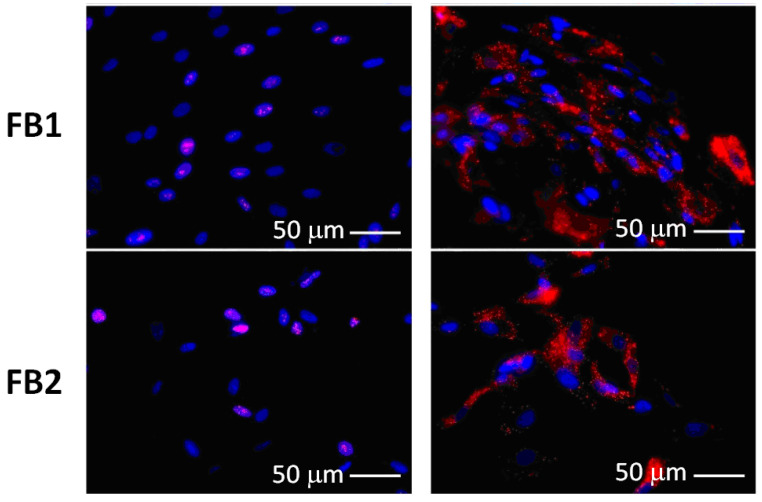
Evaluation of the rate of proliferating cells for UC MSCs (**left**) and cell morphology (**right**).

Cell adhesion for **FB1**–**FB4** was studied using dynamic method of the seeding of UC MSCs (see Section 2.5). Cell count was evaluated based on the number of viable cells, stained with DAPI, that were adhered to the film surface (Figure 7 and Figure 8, left). To observe the cell morphology, UC MSCs were labeled with a fluorescent red-orange vital dye PKH26 (Figure 8, right). Minor difference in cell adhesion was detected for **FB1** and **FB2**, but the presence of poly(EtOEP) fragments resulted in a lowering of the number of cells adhered. For copolymers with higher EtOEP content **P3** and **P4** minimal cell adhesion was detected.

**Figure 9:**
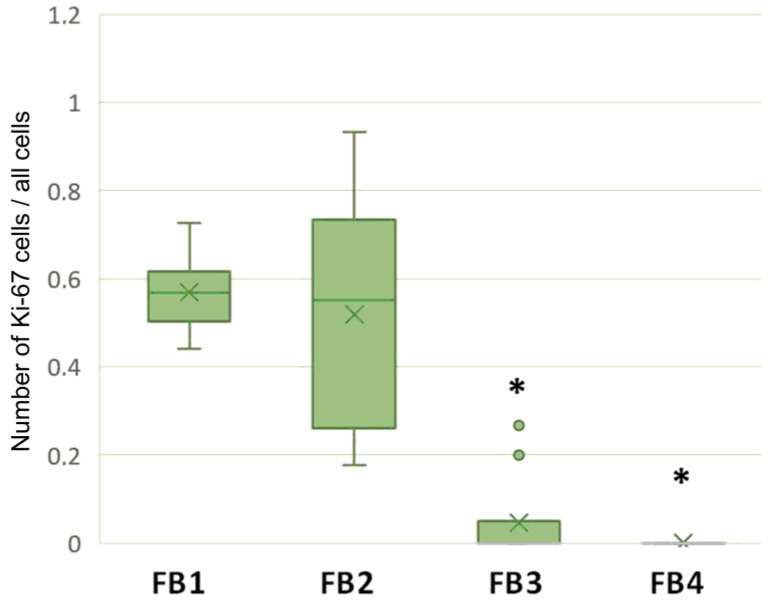
Evaluation of the proliferative properties of UC MSCs by the rate of Ki-67+ cells. * *p* < 0.05 in comparison with **FB-1**.

Immunocytochemical study showed a high level of the expression of proliferation marker Ki-67 in MSCs seeded on **FB1** and **FB2**, (Figure 8 right and Figure 9). A significantly lower rate of proliferating cells was detected for **FB3** and **FB4**.

Summarizing the results of experiments on protein adhesion, cell adhesion, and cell proliferation, it can be concluded that even ~10% mol content of poly(ethylene phosphate) leads to almost complete suppression of these processes. In this way, on the example of poly(EtOEP) the efficiency of polyphosphate-based approach to anti-adhesive materials have been demonstrated. However, in vitro studies must be supplemented by in vivo studies that are able to reveal unknown side processes accompanying the use of novel polymer implants.

### 3.7. Subcutaneous Implantation Experiments

**Figure 10:**
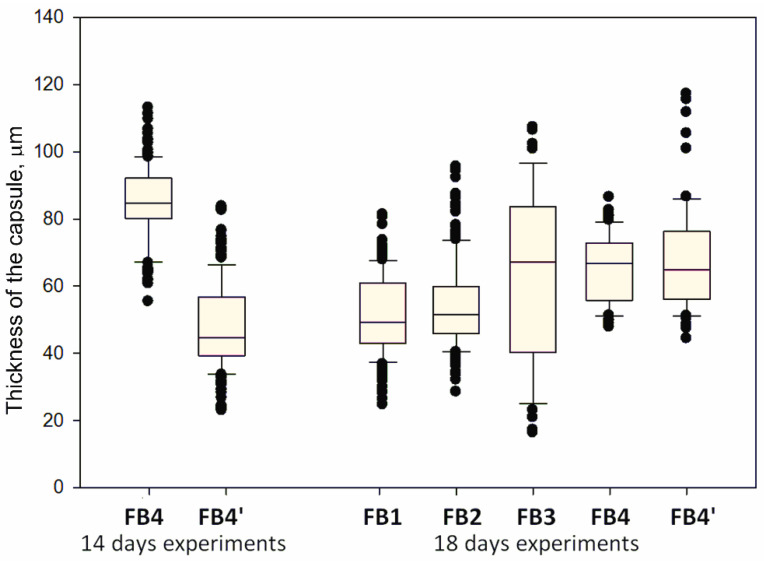
The thickness of the connective tissue capsule (μm) formed after subcutaneous implantation of the films **FB1**–**FB4** and **FB4′**.

A connective tissue capsule was formed after subcutaneous implantation of all film samples. After 14 days, the thickness of the capsule around **FB4** sample was two times higher in comparison with **FB4′**, after additional two weeks the difference has almost disappeared (Figure 10). The least thickness of the capsule was detected for **FB1** and **FB2**. Thus, despite the explicit antiadhesive properties, copolymers with relatively high EtOEP content caused a rejection reaction. It can be assumed that this reaction is caused by the toxic response to acidic products [39] of the biodegradation of EtOEP-containing copolymers.

Analysis of ^1^H NMR spectrum of **FB4** sample after 14 days of implantation (Figure 4d) showed three-quarters hydrolysis of the starting copolymer. Note that the rate of hydrolytic degradation in vivo was significantly higher than the rate of hydrolysis in PBS at the same temperature.

**Figure 11:**
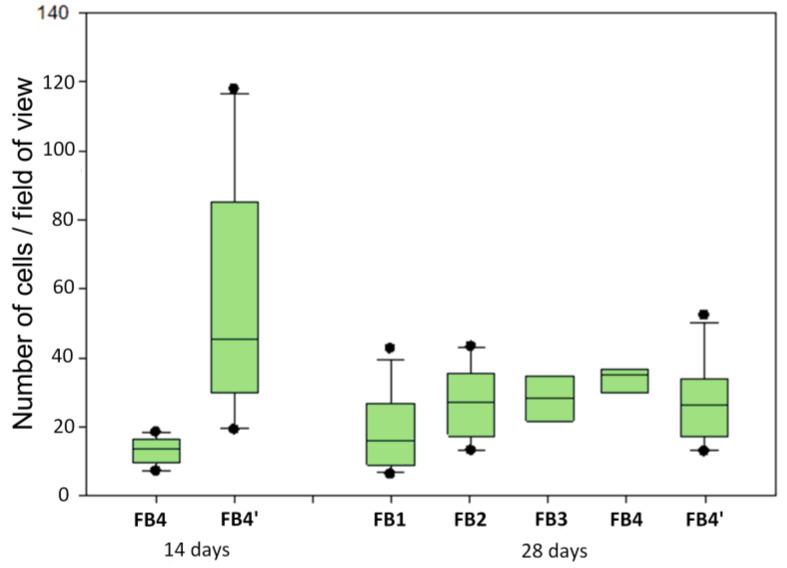
Number of CD68+ at the border of the polymer film and fibrous capsule.

To assess the immune response during subcutaneous injection of the samples, a comparative analysis of the number of CD68+ cells was performed for the film/fibrous capsule border (Figure 11, Figure S6 in the Supplementary Materials). The highest CD68+ infiltration rate was detected after 14 days for **FB4**, 45.4 (31.3–78.7) cells/mm, for **FB4′** the value of 13.5 (10.6–15.7) cells/mm was detected (Figure 11). After 28 days, **FB4** was a leader (35.4 (31.6–36.9) cells/mm), the lowest infiltration was found for **FB1** (15.6 (9.4–24.5) cells/mm) (Figure 11). Thus, immune response was also grown with the increasing of the content of EtOEP in copolymer.

## 4. Conclusions

At the beginning of the study it was suggested that poly(ethylene phosphate)s represent promising alternatives to PEG in the development of biomedical materials with antiadhesive properties. Using non-toxic BHT-Mg catalyst of coordination ROP, εCL homopolymer **P1** and three εCL/ethyl ethylene phosphate (EtOEP) block copolymers **P2**–**P4** with *DP*_n_ (εCL) ~200 and different εCL/EtOEP ratios have been prepared, and polymer films for the further studies on hydrolytic degradation and biocompatibility have been made.

It has been found that in vitro hydrolytic degradation of εCL/EtOEP block copolymers proceeds as a surface hydrolysis of polyphosphate fragments. This process was accompanied by the formation of extended caverns (peculiar ‘chemical crazing’). As expected, εCL/EtOEP block copolymers demonstrated anti-adhesive properties against proteins (GFP) and cells (MSCs). However, subcutaneous implantation experiments showed that poly(EtOEP)-containing films cause the formation of fibrous capsules, presumably due to response on the formation of acidic products during the hydrolysis of polyphosphate.

The results of these studies can be used for the further development of biocompatible and biodegradable materials for tissue engineering and other biomedical applications. In can be assumed that negative response, detected during in vivo experiments, can be mitigated by the use of basic components in formulations of the prospective polymer composites.

## Figures and Tables

**Table 1:** Polymerization experimental data for BHT-Mg catalyzed (co)polymerization. Reaction conditions: CH_2_Cl_2_/THF, [εCL] = 2 M.

Run	εCL/EtOEP/BHT-Mg Initial Ratio	εCL Conv., % ^1^	EtOEP Conv., % ^1^	Polymer Composition *DP*_n_ (εCL)/ *DP*_n_ (EtOEP) ^2^	*M_n_*^theo^ × 10^3 3^	*M_n_*^NMR^ × 10^3 2^	*M_n_*^SEC^ × 10^3 4^	*Ð_M_* ^4^
**P1^628^**	200/–/1	>99	—	—	22.9	30.0	36.0	1.38
**P2**	200/20/1	>99	83	31.6	25.2	34.9	32.7	1.81
**P3**	200/40/1	>99	92	8.3	27.9	31.3	28.8	2.00
**P4**	200/60/1	>99	87	2.8	32.4	32.6	46.5	2.04

^1^*DP*_n_ (degree of polymerization) was determined by the analysis of the NMR spectra of the reaction mixtures. ^2^ *M_n_*^theo^ = n × M(εCL) + m × M(EtOEP) + M(BnOH), where n and m are the numbers of εCL and EtOEP fragments determined by comparative integration of the signals of BnO group (δ ~7.3 ppm, 5H) and signals of CH_2_O fragments of εCL (δ = 4.05 ppm, 2nH) and EtOEP (δ = 4.15–4.30 ppm, 6mH). ^3^ *M_n_*^theo^ = M(εCL) × Conv. (εCL) + M(EtOEP) × Conv. (EtOEP) + M(BnOH). ^4^ Determined by size exclusion chromatography (SEC) in THF vs. polystyrene standards and corrected by the factor of 0.56.

**Table 2:** Tensile properties of polymer films.

Polymer	Average Cross-Sectional Area, mm^2^	Tensile Strength, MPa	Yield Stress σ, MPa	Young’s Modulus E, MPa	Elongation at Break ε_p_, %
**FM1**	0.81	20.2 ± 9.6	14.5 ± 0.6	207 ± 19	72 ± 78
**FM2**	0.84	11.9 ± 5.0	16.6 ± 0.7	251 ± 20	455 ± 147
**FM3**	0.82	9.5 ± 6.2	14.8 ± 0.5	200 ± 39	515 ± 100
**FM4**	0.82	4.0 ± 1.4	6.3 ± 2.0	163 ± 68	4.0 ± 0.8

## Supplementary Materials

The following are available online at https://www.mdpi.com/2073-4360/12/12/3039/s1, **Figure S1**. ^1^H NMR spectrum (400 MHz, CDCl_3_, 20 °C) of εCL homopolymer **P1**; **Figure S2**. ^1^H NMR spectrum (400 MHz, CDCl_3_, 20 °C) of copolymer **P2**; **Figure S3**. ^1^H NMR spectrum (400 MHz, CDCl_3_, 20 °C) of copolymer **P3**; **Figure S4**. ^1^H NMR spectrum (400 MHz, CDCl_3_, 20 °C) of copolymer **P4**; **Figure S5.** Connective tissue capsules after 28 days after subcutaneous administration of the polymer films. Stained by hematoxylin and eosin, line segment 100 μm; **Figure S6**. CD68+ cells at the border between fibrous capsule and film. Immunocytochemical stain, fluorescent microscopy, cell nuclei stained by DAPI, line segment 50 μm.
