# Supplementary material for: In Vitro and In Vivo Studies of Biodegradability and Biocompatibility of Poly(εCL)-b-Poly(EtOEP)-Based Films
Source: Polymers (Basel). 2020 Dec 18;12(12):3039. doi: 10.3390/polym12123039 (PMC7766882; doi:10.3390/polym12123039)
Supplement: Supplementary file 1 [file polymers-12-03039-s001.pdf]

In Vitro and *In Vivo* Studies of Biodegradability and Biocompatibility of poly(εCL)-*b*-poly(EtOEP)-based Films

Ilya Nifant'ev [1,2,3,*], Andrey Shlyakhtin [1], Pavel Komarov [2], Alexander Tavtorkin [2], Evgeniya Kananykhina [4], Andrey Elchaninov [5], Polina Vishnyakova [5], Timur Fatkhudinov [4,6] and Pavel Ivchenko [1,2]

[1] Chemistry Department, M.V. Lomonosov Moscow State University, 1-3 Leninskie Gory, 119991 Moscow, Russia; shlyahtinav@mail.ru (A.S.); phpasha1@yandex.ru (P.I.)
[2] A.V. Topchiev Institute of Petrochemical Synthesis RAS, 29 Leninsky Pr., 119991 Moscow, Russia; komarrikov@yandex.ru (P.K.); tavtorkin@yandex.ru (A.T.)
[3] Faculty of Chemistry, National Research University Higher School of Economics, 20 Miasnitskaya Str., 101000 Moscow, Russia
[4] Research Institute of Human Morphology, 3 Tsyurupy St., 117418 Moscow, Russia; e.kananykhina@gmail.com (E.K.); tfat@yandex.ru (T.F.)
[5] National Medical Research Center for Obstetrics Gynecology and Perinatology Named after Academician V.I. Kulakov of Ministry of Healthcare of Russian Federation, 4 Oparina Str., 117997 Moscow, Russia; elchandrey@yandex.ru (A.E.); vpa2002@mail.ru (P.V.)
[6] Faculty of Science, Peoples' Friendship University of Russia, Miklukho-Maklaya 6 Str., 117198 Moscow, Russia
* Correspondence: ilnif@yahoo.com or inif@org.chem.msu.ru; Tel.: +7-495-939-4098

SUPPORTING INFORMATION

S1. NMR spectra of (co)polymers 2

S2. Supplementary materials for the studies of polymer films 4

S1. NMR spectra of (co)polymers

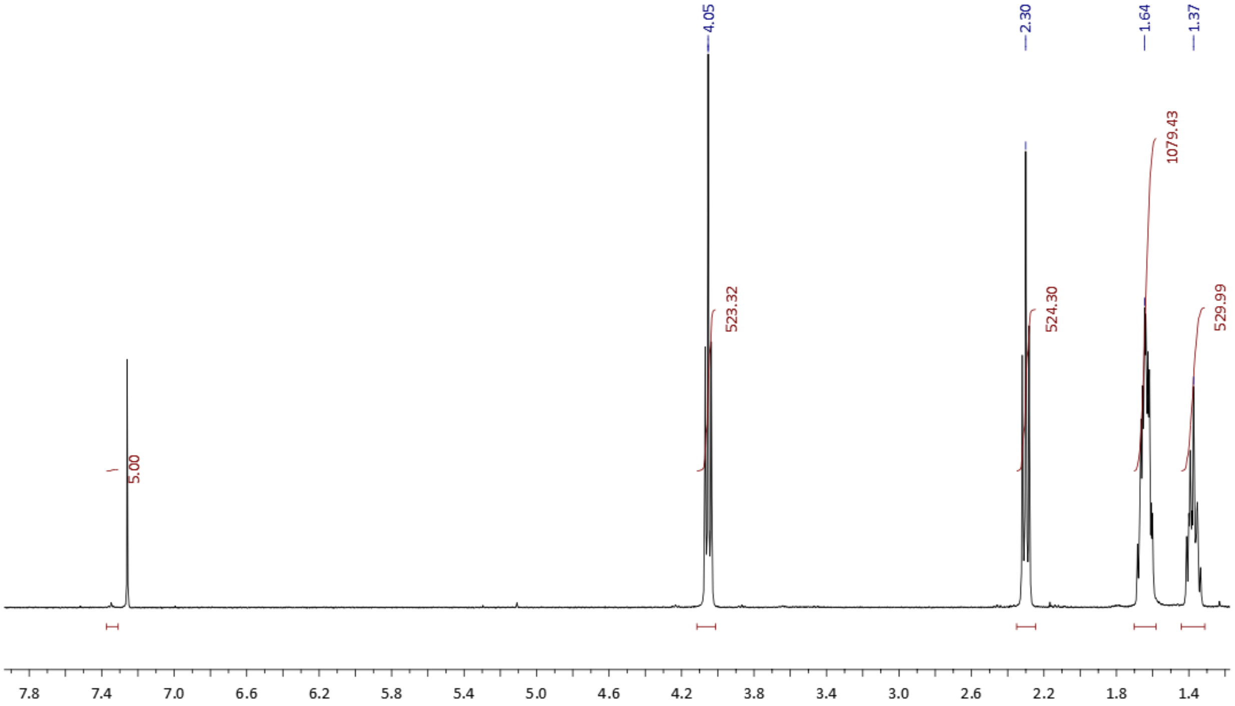

Figure S1. ^{1}H NMR spectrum (400 MHz, $CDCl_3$, 20 °C) of εCL homopolymer **P1**.

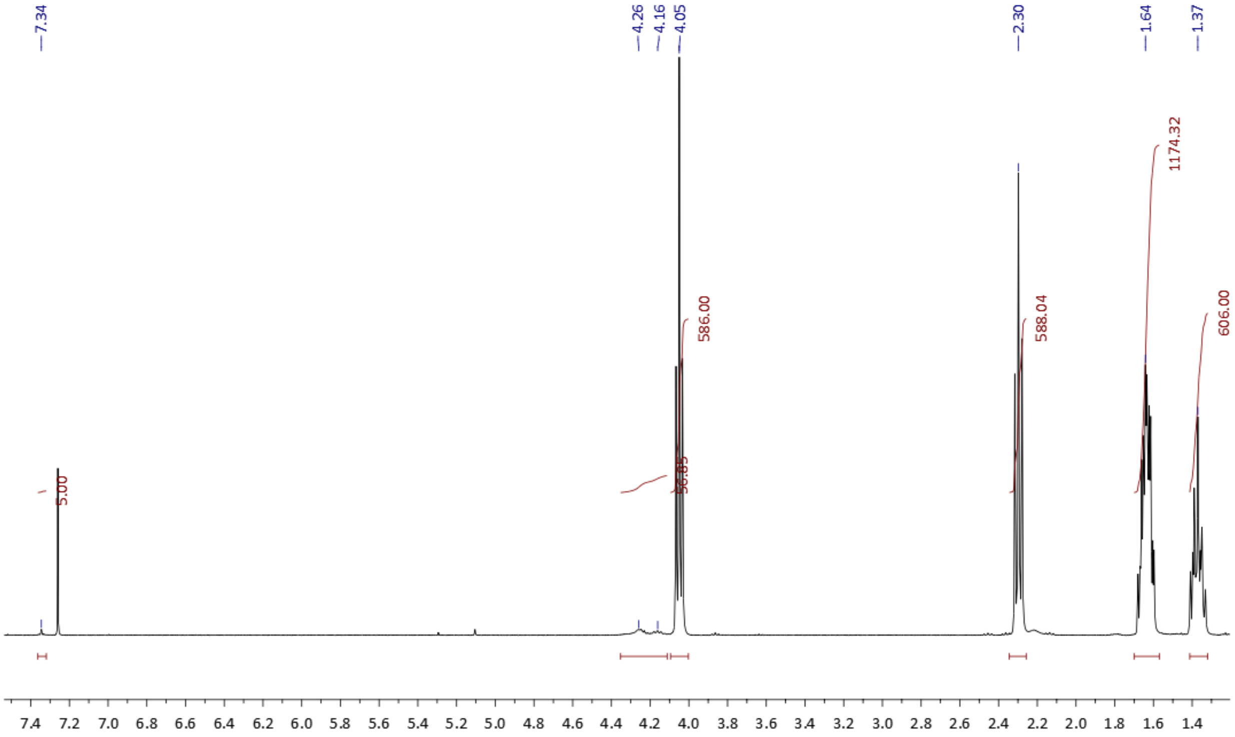

Figure S2. ^{1}H NMR spectrum (400 MHz, $CDCl_3$, 20 °C) of copolymer **P2**.

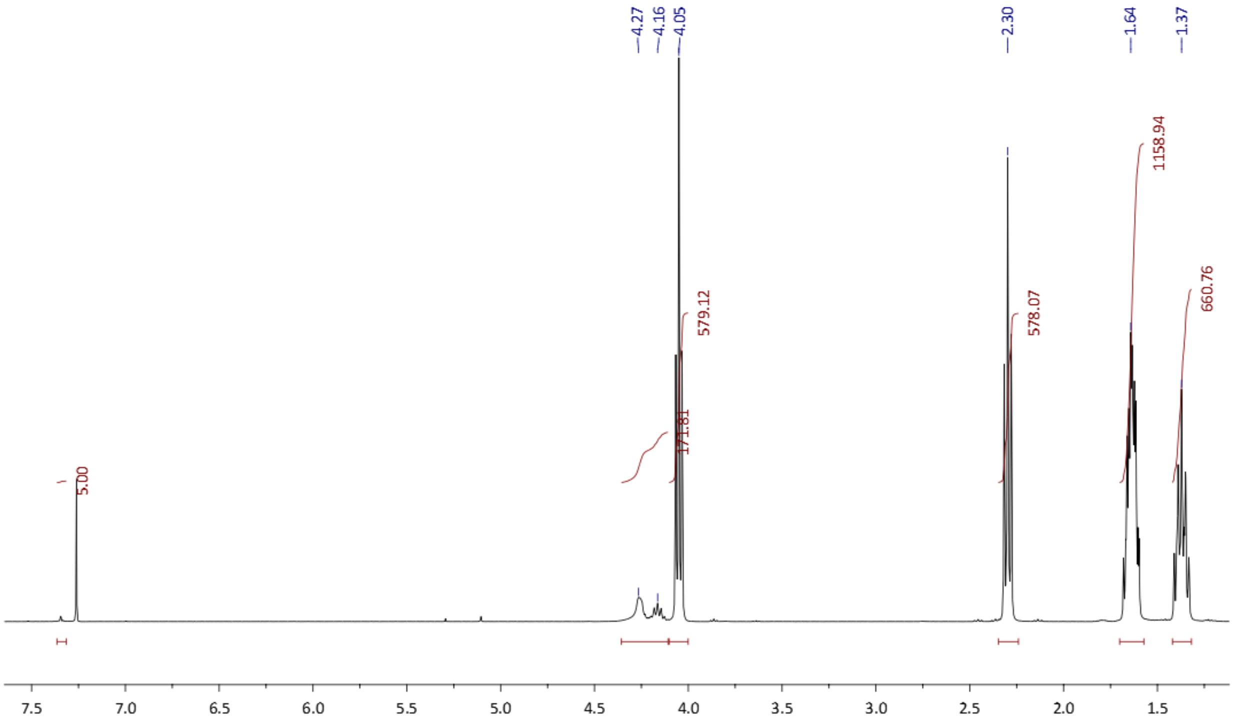

Figure S3. ^{1}H NMR spectrum (400 MHz, $CDCl_3$, 20 °C) of copolymer **P3**.

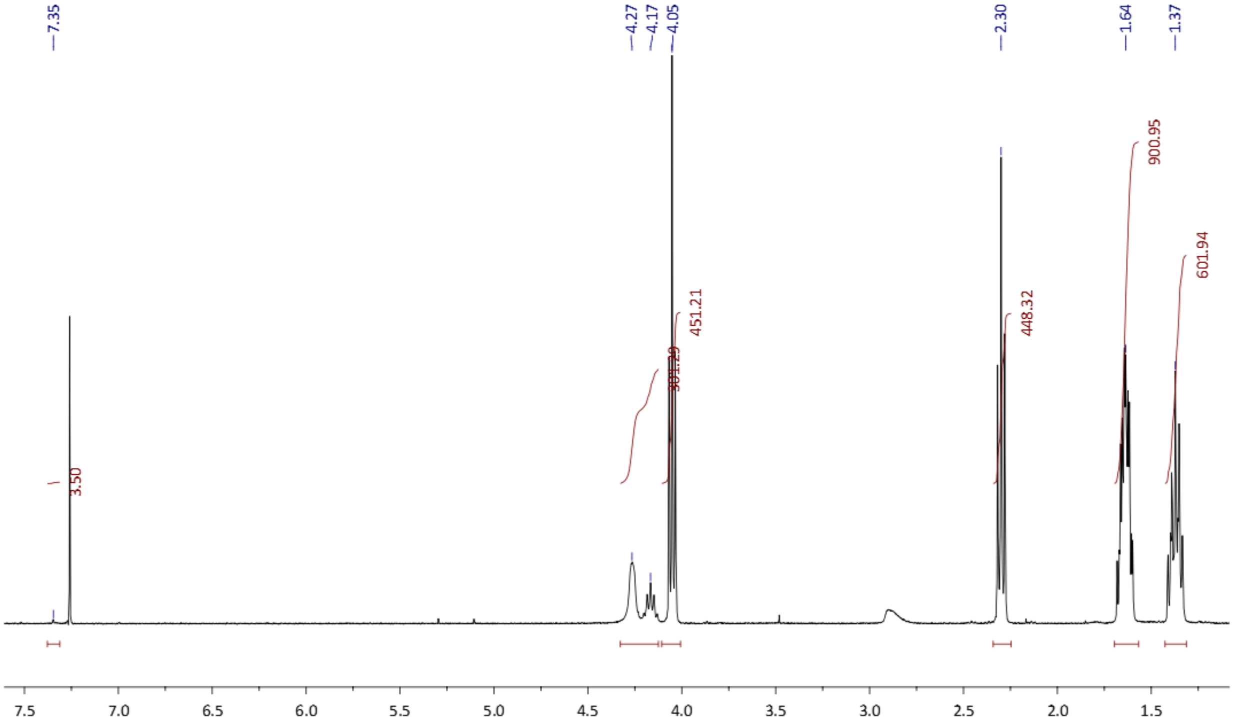

Figure S4. ^{1}H NMR spectrum (400 MHz, $CDCl_3$, 20 °C) of copolymer **P4**.

S2. Supplementary materials for the studies of polymer films

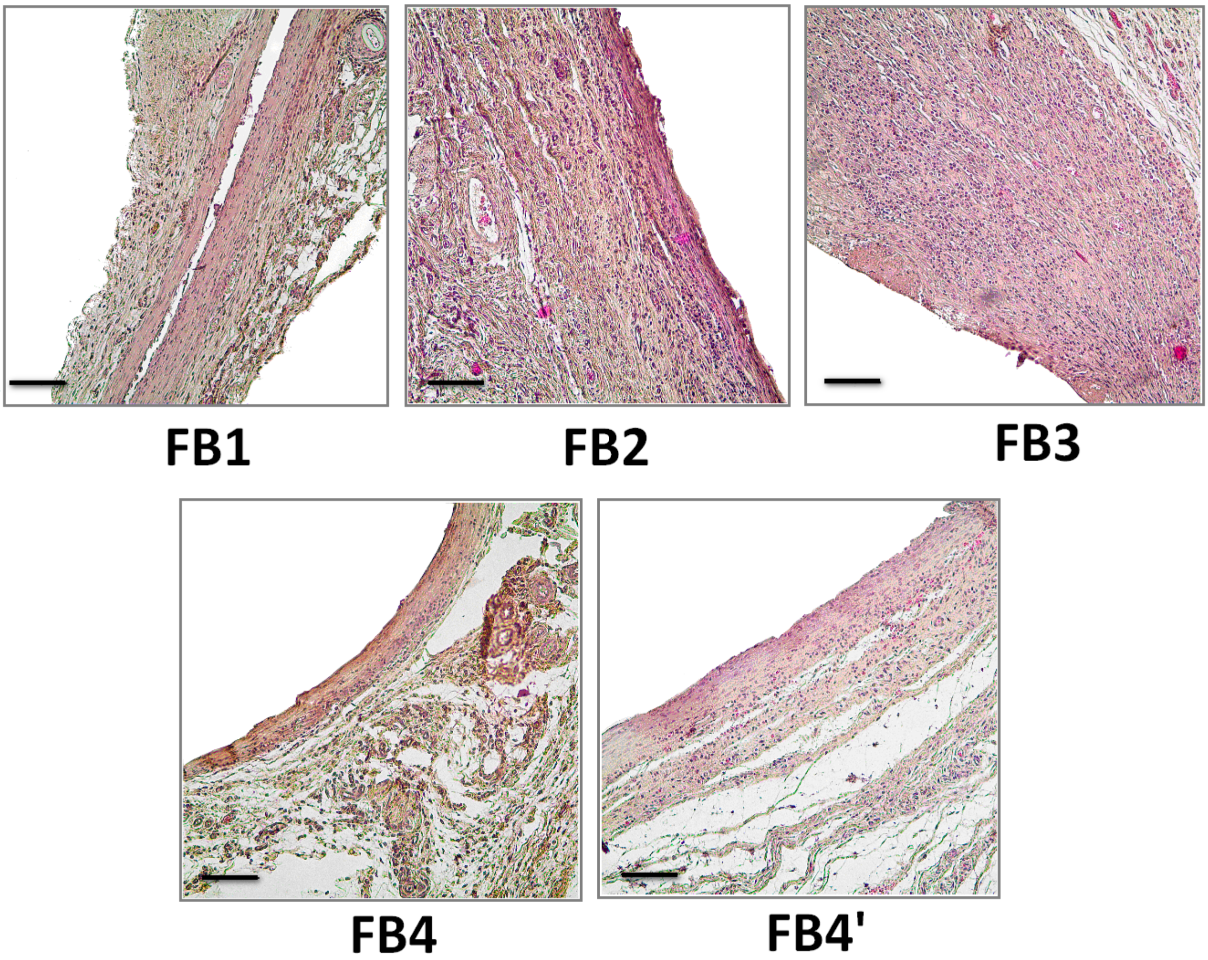

Figure S5. Connective tissue capsules after 28 days after subcutaneous administration of the polymer films. Stained by hematoxylin and eosin, line segment 100 µm.

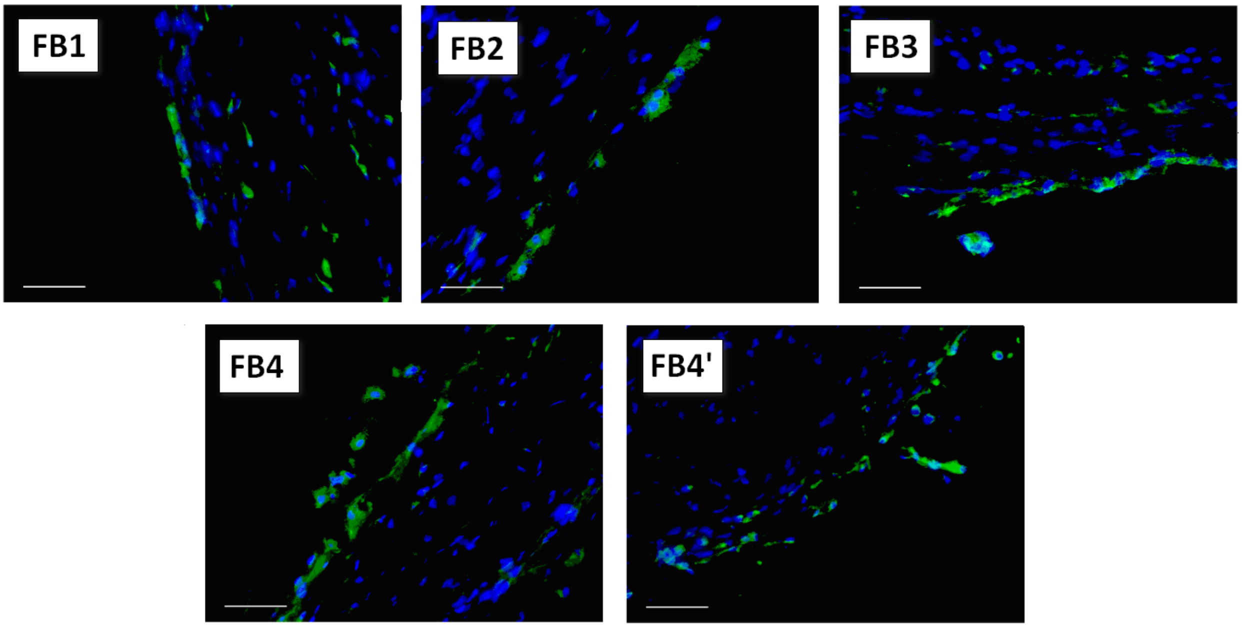

Figure S6. CD68+ cells at the border between fibrous capsule and film. Immunocytochemical stain, fluorescent microscopy, cell nuclei stained by DAPI, line segment 50 µm.
